# Complete genome sequence of *Streptobacillus moniliformis* type strain (9901^T^)

**DOI:** 10.4056/sigs.48727

**Published:** 2009-12-30

**Authors:** Matt Nolan, Sabine Gronow, Alla Lapidus, Natalia Ivanova, Alex Copeland, Susan Lucas, Tijana Glavina Del Rio, Feng Chen, Hope Tice, Sam Pitluck, Jan-Fang Cheng, David Sims, Linda Meincke, David Bruce, Lynne Goodwin, Thomas Brettin, Cliff Han, John C. Detter, Galina Ovchinikova, Amrita Pati, Konstantinos Mavromatis, Natalia Mikhailova, Amy Chen, Krishna Palaniappan, Miriam Land, Loren Hauser, Yun-Juan Chang, Cynthia D. Jeffries, Manfred Rohde, Cathrin Spröer, Markus Göker, Jim Bristow, Jonathan A. Eisen, Victor Markowitz, Philip Hugenholtz, Nikos C. Kyrpides, Hans-Peter Klenk, Patrick Chain

**Affiliations:** 1 DOE Joint Genome Institute, Walnut Creek, California, USA; 2 DSMZ - German Collection of Microorganisms and Cell Cultures GmbH, Braunschweig, Germany; 3 Los Alamos National Laboratory, Bioscience Division, Los Alamos, New Mexico, USA; 4 Biological Data Management and Technology Center, Lawrence Berkeley National Laboratory, Berkeley, California, USA; 5 Oak Ridge National Laboratory, Oak Ridge, Tennessee, USA; 6 HZI - Helmholtz Centre for Infection Research, Braunschweig, Germany; 7 University of California Davis Genome Center, Davis, California, USA

**Keywords:** *Fusobacteria*, '*Leptotrichiaceae*', Gram-negative, rods in chains, L-form, zoonotic disease, non-motile, non-sporulating, facultative anaerobic, Tree of Life

## Abstract

*Streptobacillus moniliformis* Levaditi *et al.* 1925 is the type and sole species of the genus *Streptobacillus*, and is of phylogenetic interest because of its isolated location in the sparsely populated and neither taxonomically nor genomically much accessed family '*Leptotrichiaceae*' within the phylum *Fusobacteria*. The '*Leptotrichiaceae*' have not been well characterized, genomically or taxonomically. *S. moniliformis,*is a Gram-negative, non-motile, pleomorphic bacterium and is the etiologic agent of rat bite fever and Haverhill fever. Strain 9901^T^, the type strain of the species, was isolated from a patient with rat bite fever. Here we describe the features of this organism, together with the complete genome sequence and annotation. This is only the second completed genome sequence of the order *Fusobacteriales* and no more than the third sequence from the phylum *Fusobacteria*. The 1,662,578 bp long chromosome and the 10,702 bp plasmid with a total of 1511 protein-coding and 55 RNA genes are part of the *** G****enomic* *** E****ncyclopedia of* *** B****acteria and* *** A****rchaea * project.

## Introduction

Strain 9901^T^ (= DSM 12112 = ATCC 14647 = NCTC 10651) is the type strain of *Streptobacillus moniliformis*, which also represents the type species of the genus first described in 1925 by Levaditi *et al.* [1,2] The taxonomic history of *S. moniliformis*; affiliated several genera such as '*Haverhillia* [1]' and was only placed recently in the family “*Leptotrichiaceae”* (unpublished). It has also been suggested that *S. moniliformis* be placed within the *Mycoplasmatales* due to its similarity to some members based on the low G+C content of 24-26%, the fastidious requirements for growth and the production of L-form organisms [3]. *S. moniliformis* is commonly found in the nasopharynx of feral rats as well as in laboratory or pet rats. Between 50 and 100% of wild rats carry the commensal and secrete it with their urine [4]. The organism has been associated with rat bite fever and Haverhill fever in humans, following a bite or contamination of food by rat urine, respectively. Before it could be demonstrated that both diseases are caused by the same organism, the etiologic agent for Haverhill fever was called '*Haverhillia multiformis*' [5]. Both are systemic illnesses characterized by fever, rigors and migratory polyarthralgias and nearly 75% of patients develop a rash. Untreated, rat bite fever has a mortality rate of approximately 10%, with most deaths occurring due to endocarditis [6].

*S. moniliformis* is only the second species from the phylum *Fusobacteria* for which a complete genome sequence is described. Here we present a summary classification and a set of features for *S. moniliformis* strain 9901^T^ (Table 1), together with the description of the complete genomic sequencing and annotation.

**Table 1:** Classification and general features of *S. moniliformis* 9901^T^ in accordance to the MIGS recommendations [7]

**MIGS ID**	**Property**	**Term**	**Evidence code**
	Current classification	Domain *Bacteria*	TAS [8]
		Phylum *Fusobacteria*	TAS [9]
		Class *Fusobacteria*	TAS [9]
		Order *Fusobacteriales*	TAS [9]
		Family *'Leptotrichiaceae'*	NAS
		Genus *Streptobacillus*	TAS [1]
		Species *Streptobacillus moniliformis*	TAS [1]
		Type strain 9901	TAS [1]
	Gram stain	negative	TAS [1]
	Cell shape	long rods	TAS [1]
	Motility	nonmotile	TAS [1]
	Sporulation	non-sporulating	TAS [1]
	Temperature range	mesophile	TAS [1]
	Optimum temperature	37°C	TAS [1]
	Salinity	normal	TAS [1]
MIGS-22	Oxygen requirement	facultative anaerobic	TAS [1]
	Carbon source	monosaccharides, starch	TAS [10]
	Energy source	carbohydrates	TAS [10]
MIGS-6	Habitat	nasopharynx of rats	TAS [1]
MIGS-15	Biotic relationship	free living	NAS
MIGS-14	Pathogenicity	pathogenic for humans	TAS [1]
	Biosafety level	2	TAS [11]
	Isolation	patient with rat-bite fever	NAS
MIGS-4	Geographic location	France	NAS
MIGS-5	Sample collection time	unknown	
MIGS-4.1 MIGS-4.2	Latitude , Longitude	unknown	
MIGS-4.3	Depth	not reported	
MIGS-4.4	Altitude	not reported	

Evidence codes - IDA: Inferred from Direct Assay (first time in publication); TAS: Traceable Author Statement (i.e., a direct report exists in the literature); NAS: Non-traceable Author Statement (i.e., not directly observed for the living, isolated sample, but based on a generally accepted property for the species, or anecdotal evidence). These evidence codes are from the Gene Ontology project [12]. If the evidence code is IDA, then the property was observed for a living isolate by one of the authors or an expert mentioned in the acknowledgements.

## Classification and features

Isolate H2730, from a clinical case of fatal rat bite fever in the US [13] perfectly matches the 16S rRNA gene sequence of the genome of strain 9901^T^ described in this report; other recently described strains (TSD4, IKB1, IKC1, and IKC5) isolated from feral rats in Japan differ in just 1-4 nucleotides [14]. No phylotypes from environmental screening or genomic surveys could be linked with more than 90% 16S rRNA sequence similarity to *S. moniliformis* (status May 2009), indicating that the strain is rarely found in the environment outside of its natural hosts.

**Figure 1:**
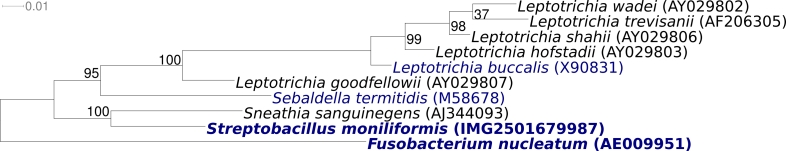
Phylogenetic tree highlighting the position of *S. moniliformis* 9901^T^ relative to the other type strains of the family ‘*Leptotrichiaceae*’. The tree was inferred from 1399 aligned characters [15,16] of the 16S rRNA sequence under the maximum likelihood criterion [17] and rooted with the type strain of the family '*Fusobacteriaceae*' The branches are scaled in terms of the expected number of substitutions per site. Numbers above branches are support values from 1,000 bootstrap replicates if larger than 60%. Lineages with type strain genome sequencing projects registered in GOLD [18] are shown in blue, published genomes in bold, *e.g.* the GEBA type strain *Leptotrichia buccalis* [19].

Figure 1 shows the phylogenetic neighborhood of *S. moniliformis* strain 9901^T^ in a 16S rRNA based tree. The sequences of the five 16S rRNA gene copies in the genome of *S. moniliformis* 9901^T^ do not differ from each other, and differ by six nucleotides from the previously published 16S rRNA sequence generated from ATCC 14647 (Z35305). The difference between the genome data and the reported 16S rRNA gene sequence is most likely due to sequencing errors in the previously reported sequence data.

**Figure 2:**
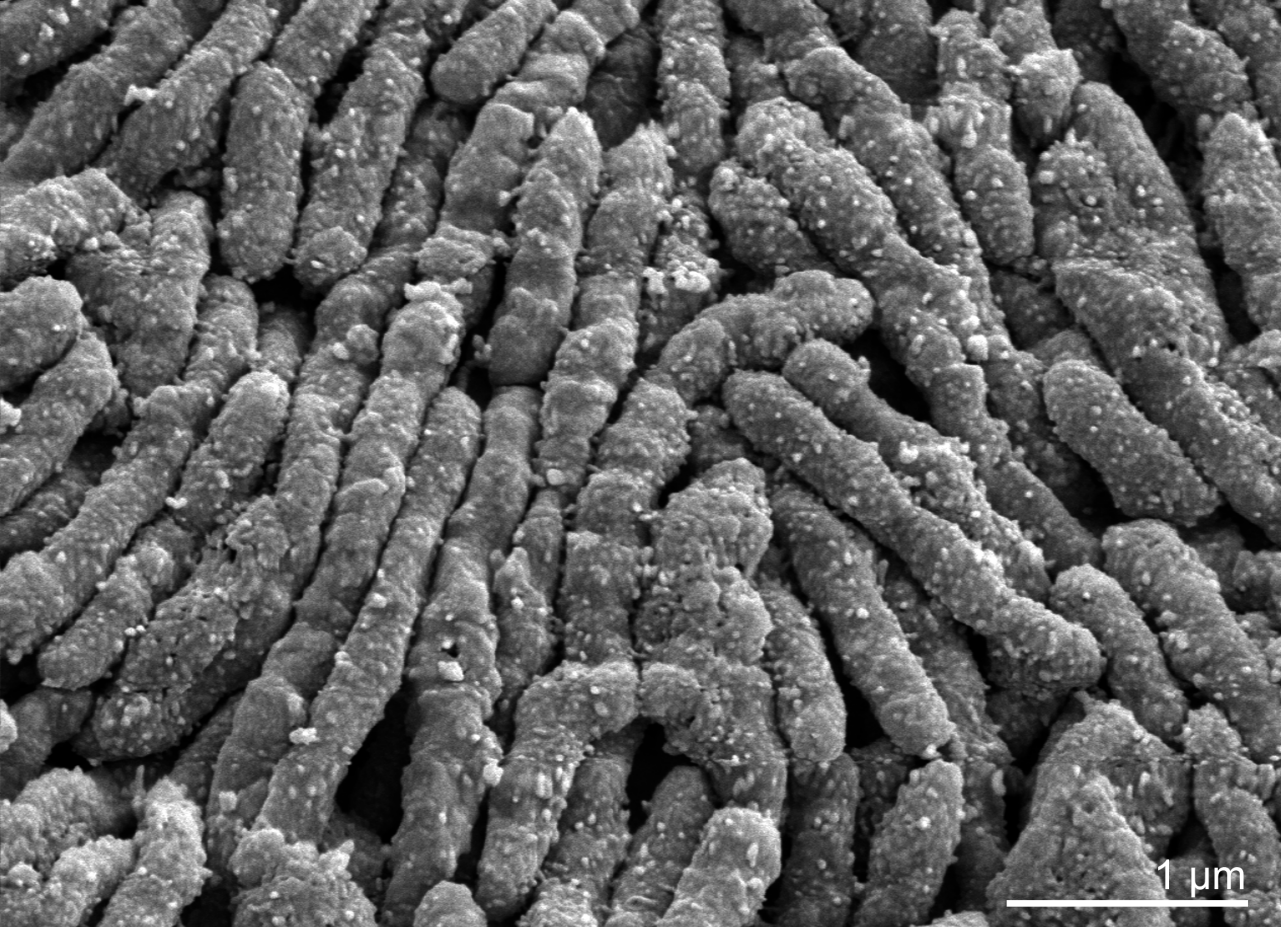
Scanning electron micrograph of *S. moniliformis* 9901^T^

*S. moniliformis* is a Gram-negative, non-motile, fastidious, slow-growing and facultatively anaerobic organism that grows as elongated rods (0.3-0.7 µm by 1-5 µm in length) which tend to form chains or filaments with occasional bulbar swellings leading to a necklace-like appearance ("moniliformis" means necklace-shaped) (Figure 2). The organism exists in two variants: the bacillary form and a cell wall-deficient L-form, which is considered nonpathogenic [20]. The primary habitat of *S. moniliformis* is small rodents, including rats (dominant reservoir) and more rarely gerbils, squirrels and mice. Rat-eating carnivores such as dogs, cats, ferrets and pigs can also become hosts and thus transfer the pathogen to humans. However, the organism is not directly transmitted from person to person and thus presents a typical zoonotic agent. A large number of case reports of *S. moniliformis* infections have been published (references in [4]). For cultivation, complex media containing blood, serum or ascitis fluid are necessary and increased CO_2_ concentration enhances growth. The organism is extremely sensitive to sodium polyanethol sulfonate ("Liquoid"), an anticoagulant used in commercial blood culture bottles, which can lead to problems during primary isolation [21]. *S. moniliformis* is catalase and oxidase negative and is biochemically rather inert. The metabolism is fermentative. Acid but no gas is produced from glucose, fructose, maltose and starch; H_2_S is produced. Arginine dihydrolase is synthesized [22,23]. *S. moniliformis* is susceptible to all β-lactam antibiotics, no β-lactamase activity could be demonstrated thus far [10].

### Chemotaxonomy

No data are available about the murein composition of strain 9901^T^. The fatty acid pattern of *S. moniliformis* can be used for its rapid identification and comprises a mixture of saturated and unsaturated straight-chain acids: C_16:0_, C_18:0_, C_18:1_ and C_18:2_. The type of menaquinones and polar lipids used by *S. moniliformis* has not been described yet.

## Genome sequencing and annotation

### Genome project history

This organism was selected for sequencing on the basis of its phylogenetic position, and is part of the *** G****enomic* *** E****ncyclopedia of* *** B****acteria and* *** A****rchaea * project. The genome project is deposited in the Genomes OnLine Database [18] and the complete genome sequence in GenBank. Sequencing, finishing and annotation were performed by the DOE Joint Genome Institute (JGI). A summary of the project information is shown in Table 2.

**Table 2:** Genome sequencing project information

**MIGS ID**	**Property**	**Term**
MIGS-31	Finishing quality	Finished
MIGS-28	Libraries used	Two genomic libraries: 8kb pMCL200 and fosmid pcc1Fos Sanger libraries. One 454 pyrosequence standard library
MIGS-29	Sequencing platforms	ABI3730, 454 GS FLX
MIGS-31.2	Sequencing coverage	11.5× Sanger; 24.9x pyrosequence
MIGS-30	Assemblers	Newbler version 1.1.02.15, phrap
MIGS-32	Gene calling method	Prodigal 1.4, GenePRIMP
	INSDC / Genbank ID	CP001779
	Genbank Date of Release	November 19, 2009
	GOLD ID	Gc01145
	NCBI project ID	29309
	Database: IMG-GEBA	2501651197
MIGS-13	Source material identifier	DSM 12112
	Project relevance	Tree of Life, GEBA, Medical

### Growth conditions and DNA isolation

*S. moniliformis* strain 9901^T^, DSM 12112, was grown aerobically with high humidity and increased CO_2_ concentration on DSMZ medium 429 (Columbia Blood Agar [24] at 37°C. DNA was isolated from 0.4 g of cell paste using Qiagen Genomic 500 DNA Kit (Qiagen, Hilden, Germany) following the manufacturer's instructions, but with cell lysis modification ‘L’ solution according to Wu *et al* [25].

### Genome sequencing and assembly

The genome was sequenced using a combination of Sanger and 454 sequencing platforms. All general aspects of library construction and sequencing performed at the JGI can be found at the JGI website (http://www.jgi.doe.gov). 454 Pyrosequencing reads were assembled using the Newbler assembler version 1.1.02.15 (Roche). Large Newbler contigs were broken into 1,459 overlapping fragments of 1,000 bp and entered into assembly as pseudo-reads. The sequences were assigned quality scores based on Newbler consensus q-scores with modifications to account for overlap redundancy and to adjust inflated q-scores. A hybrid 454/Sanger assembly was made using the phrap assembler (High Performance Software, LLC). Possible mis-assemblies were corrected with Dupfinisher or transposon bombing of bridging clones [26]. Gaps between contigs were closed by editing in Consed, custom primer walk or PCR amplification. 1,081 Sanger finishing reads were produced to close gaps and to raise the quality of the finished sequence. The error rate of the completed genome sequence is less than 1 in 100,000. The final assembly consists of 22,979 Sanger and 326,576 pyrosequence reads. Together all sequence types provided 36.4× coverage of the genome.

### Genome annotation

Genes were identified using Prodigal [27] as part of the Oak Ridge National Laboratory genome annotation pipeline, followed by a round of manual curation using the JGI GenePRIMP pipeline (http://geneprimp.jgi-psf.org) [28]. The predicted CDSs were translated and used to search the National Center for Biotechnology Information (NCBI) non-redundant database, UniProt, TIGRFam, Pfam, PRIAM, KEGG, COG, and InterPro databases. Additional gene prediction analysis and functional annotation was performed within the Integrated Microbial Genomes - Expert Review (IMG-ER) platform (http://img.jgi.doe.ogv/er) [29].

## Genome properties

**Figure 3:**
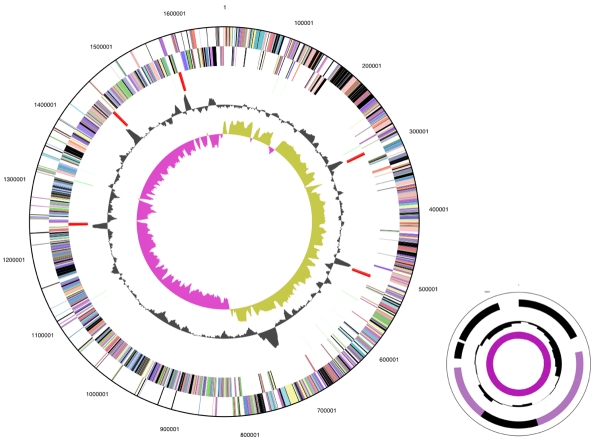
Graphical circular map of the genome. Lower-right part: plasmid, not drawn to scale. From outside to the center: Genes on forward strand (color by COG categories), Genes on reverse strand (color by COG categories), RNA genes (tRNAs green, rRNAs red, other RNAs black), GC content, GC skew.

The genome is 1,673,280 bp long and comprises one circular chromosome and one plasmid with a 26.3% GC content (Table 3 and Figure 3). Of the 1,566 genes predicted, 1,511 were protein coding genes, and 55 RNAs. A total of 69 pseudogenes were also identified. The majority of the protein-coding genes (67.3%) genes were assigned with a putative function, while the remaining ones were annotated as hypothetical proteins. The properties and the statistics of the genome are summarized in Table 3. The distribution of genes into COGs functional categories is presented in Table 4.

**Table 3:** Genome Statistics

**Attribute**	**Value**	**% of Total**
Genome size (bp)	1,673,280	100.00%
DNA coding region (bp)	1,556,870	93.04%
DNA G+C content (bp)	439,733	26.28%
Number of replicons	2	
Extrachromosomal elements	1	
Total genes	1,566	100.00%
RNA genes	55	3.51%
rRNA operons	5	
Protein-coding genes	1,511	96.49%
Pseudo genes	69	4.41%
Genes with function prediction	1,054	67.31%
Genes in paralog clusters	321	20.50%
Genes assigned to COGs	1,018	65.01%
Genes assigned Pfam domains	1,067	68.18%
Genes with signal peptides	262	16.73%
Genes with transmembrane helices	343	21.90%
CRISPR repeats	1	

**Table 4:** Number of genes associated with the general COG functional categories

Code	value	% age	Description
J	134	8.9	Translation, ribosomal structure and biogenesis
A	2	0.1	RNA processing and modification
K	66	4.4	Transcription
L	88	5.8	Replication, recombination and repair
B	0	0.0	Chromatin structure and dynamics
D	18	1.2	Cell cycle control, mitosis and meiosis
Y	0	0.0	Nuclear structure
V	32	2.1	Defense mechanisms
T	25	1.7	Signal transduction mechanisms
M	57	3.8	Cell wall/membrane biogenesis
N	11	0.7	Cell motility
Z	0	0.0	Cytoskeleton
W	2	0.1	Extracellular structures
U	36	2.4	Intracellular trafficking and secretion
O	45	3.0	Posttranslational modification, protein turnover, chaperones
C	39	2.6	Energy production and conversion
G	119	7.9	Carbohydrate transport and metabolism
E	75	5.0	Amino acid transport and metabolism
F	50	3.3	Nucleotide transport and metabolism
H	21	1.4	Coenzyme transport and metabolism
I	25	1.7	Lipid transport and metabolism
P	56	3.7	Inorganic ion transport and metabolism
Q	4	0.3	Secondary metabolites biosynthesis, transport and catabolism
R	114	7.5	General function prediction only
S	72	4.8	Function unknown
-	493	32.6	Not in COGs
